# Vector-borne disease risk indexes in spatially structured populations

**DOI:** 10.1371/journal.pntd.0006234

**Published:** 2018-02-12

**Authors:** Jorge Velázquez-Castro, Andrés Anzo-Hernández, Beatriz Bonilla-Capilla, Moisés Soto-Bajo, Andrés Fraguela-Collar

**Affiliations:** 1 Facultad de Ciencias Físico-Matemáticas, Benemérita Universidad Autónoma de Puebla, Puebla, México; 2 Cátedras CONACYT - Benemérita Universidad Autónoma de Puebla - Facultad de Ciencias Físico-Matemáticas, Benemérita Universidad Autónoma de Puebla, Puebla, México; University of Washington, UNITED STATES

## Abstract

There are economic and physical limitations when applying prevention and control strategies for urban vector borne diseases. Consequently, there are increasing concerns and interest in designing efficient strategies and regulations that health agencies can follow in order to reduce the imminent impact of viruses like Dengue, Zika and Chikungunya. That includes fumigation, abatization, reducing the hatcheries, picking up trash, information campaigns. A basic question that arise when designing control strategies is about which and where these ones should focus. In other words, one would like to know whether preventing the contagion or decrease vector population, and in which area of the city, is more efficient. In this work, we propose risk indexes based on the idea of secondary cases from patch to patch. Thus, they take into account human mobility and indicate which patch has more chance to be a corridor for the spread of the disease and which is more vulnerable, *i.e.* more likely to have cases?. They can also indicate the neighborhood where hatchery control will reduce more the number of potential cases. In order to illustrate the usefulness of these indexes, we run a set of numerical simulations in a mathematical model that takes into account the urban mobility and the differences in population density among the areas of a city. If we label by *i* a particular neighborhood, the transmission risk index (*TR*_*i*_) measures the potential secondary cases caused by a host in that neighborhood. The vector transmission risk index (*VTR*_*i*_) measures the potential secondary cases caused by a vector. Finally, the vulnerability risk index (*VR*_*i*_) measures the potential secondary cases in the neighborhood. Transmission indexes can be used to give geographical priority to some neighborhoods when applying prevention and control measures. On the other hand, the vulnerability index can be useful to implement monitoring campaigns or public health investment.

## Author summary

A disease is called vector-borne when it is not transmitted directly among humans, but in a human-vector-human way. Examples of major importance, due to its epidemiological magnitude, are Dengue, Chikungunya and Zika tropical diseases, for which the main vector is the *Aedes aegypti* mosquito. Usually, some indexes are used to measure the potential damage that such a disease could cause, which concern infectivity and entomological issues, human mobility and geographical density, etc.

We consider a mathematical epidemiological model which takes specifically into account these spatial factors (mobility, density), and propose a set of new risk indexes which give information about how the epidemic spread could occur, and where the outbreaks could take place. These indexes come from a preventive perspective, and they also pay attention to the epidemic dynamics. Consequently, they potentially allow to proceed in the correct places, and before troubles have arisen. We think these new tools could eventually help health agencies in the design of efficient and effective strategies on prevention and control of vector-borne diseases epidemics.

## Introduction

Dengue, Zika and Chikungunya are deseases of major concern in many countries [1, 2]. In particular, Dengue is of major importance due to its epidemiological magnitude [3, 4]. On the other hand, in many countries Zika and Chikungunya can be considered as new diseases for which a great portion of the population is susceptible. This feature make them a major epidemiological threat.

These three deseases are vector borne, transmitted by the bite of *Aedes aegypti* mosquito [1, 5]. Thus the greatest efforts in prevention and control are in reducing the mosquito population. This can be done by spying or by reducing the hatcheries. The *A. aegypti* is a domestic species in the sense that it oviposit its eggs in human made containers with clean water, so it is principally found around houses [4, 6]. The containers used by the mosquitoes for reproducing can be trash in yards or streets filled with water from rain, drums that some people use to accumulate water in case it goes scarce, or even pools and swimming pools [7]. In addition to fumigation, other strategies has been implemented to control vector reproduction capacity, like picking up unprocessed trash and abatization. The implementation of information campaigns like promoting to cap water recipients, the use of repellents and mosquito nets, or even visit the physician at first symptoms have been also important.

Implementing this measures cost money, so in big to medium cities is not normally possible to apply them in all the urban area, and a lot less in a whole country. Thus, local governments are forced to take a decision about which measures to implement with the resources at hand and where they are going to be implemented [8]. Thus, in the process to better manage the resources they always face the following questions: Where should control measures be implemented? Which kind of measures should be implemented in order to get the best impact in controlling the dispersion of the disease?

Nowadays, the traditional control and preventive measures are based on population indexes [5, 9, 10]. That means that if the larvae density is higher than a certain empirically pre-established threshold, alert is raised, and in case there are enough resources, hatchery control is carried out. These traditional indexes used by entomologists have the following disadvantages. First, they are reactive and not preventive, because of the measures are taken once a high density of mosquitoes population is detected and nothing is done to prevent its growth before that. Second, even if vector density is one of the main factors promoting the disease transmission, this kind of indexes overlook other important factors like human density and mobility [11, 12].

Human mobility and density play an important role not only in outbreaks but also in the maintenance of endemic states [12–16]. Thus, either an strategy to reduce the impact of an outbreak or an strategy to eliminate an endemic situation should take into account these human factors [17, 18] (See [19] to look some current methods to prevent malaria disease importation in countries by classifying human movement in: people in the eliminating region, during transit, in the endemic region, and upon return to the eliminating country.). This is also true because of these kind of indexes do not take into account the dynamics of the disease that is determined by the transmission mechanisms [20–23].

In this work, we derive risk indexes based in the idea of secondary cases. Then, we obtain explicit expressions for them in the frame of a dynamical mathematical model for the spread of *Aedes aegypti* borne diseases. We propose a model within a meta-population framework with a Lagrangian approach in order to take into account human intra-urban mobility. The proposed indexes arise in a natural way from the dynamical model using the theory of dynamical systems and networks [14, 24–28].

We focus our attention in two types of indexes. One of them, is a measure of the potential transmission and spread of the disease caused by human behavior. The other kind of index is a measure of the transmission caused by the particular spatial distribution of mosquitoes. Specifically, information campaigns targeted at promoting some convenient behaviors like going to hospitals at first symptoms, use of repellents, mosquito nets and large cloths can be guided by the human transmission risk index (*TR*_*i*_). This is so, because this transmission risk index is a measure of the potential secondary infections the inhabitants of a particular neighborhood cause in the whole system. On the other hand, abatization, fumigation and hatchery elimination targeting at reducing vector population can be guided by the vector transmission risk index (*VTR*_*i*_). This index is a measure of the potential secondary cases that the mosquitoes of a certain neighborhood cause in the system. Thus, this set of indexes can be used to guide a complex strategy in which the resources focused in changing vector population or human habits do not necessarily are applied in the same place. In addition to this, we also find useful the vulnerability risk index (*VR*_*i*_) to guide monitoring actions of early warning protocols as this index measures the potential secondary cases in a particular neighborhood caused by the whole system.

Even if this indexes can be used to guide control measures they are build to guide prevention measures, thus they are independent of an outbreak state. This is a main difference between the proposed risk indexes and optimal control strategies where the state of the epidemics needs to be known thus being reactive and not preventive.

While the proposed indexes consider population densities, they also take into account human mobility, so they can be used to propose a global control strategy. That means that the risk indexes take into account that a neighborhood with important human inward flux and outward flux has more chance to be a corridor for the spread of the disease. Thus, the kind of questions that can be answered using these indexes are: Which area is more likely to act as a corridor and which is more vulnerable, *i.e.* more likely to have cases? In which neighborhood the hatchery control will reduce more the number of potential cases? In which neighborhood will the resource allocation be more effective in surveillance of cases and immediate attention?

## Materials and methods

### Risk indexes

We look for to define summary measures that capture important information of how a virus transmitted by a vector (like the *A. aegypty* mosquito) is spread in a region. Let Ω be the spatial region where the vector-borne disease transmission will be analyzed. Such region could be a city or a village which can be divided into *N* disjoint subregions Ω_*i*_, such as neighborhoods, which we call patches. Thus, $\Omega =\cup_{i=1}^{N}\Omega_{i}$. Hosts and vectors coexist, but each patch has its particular social and ecological features, *i.e.* human population, transportation and mobility habits of the inhabitants, and mosquitoes density can differ from one neighborhood to other.

Because of the *A. aegypty* has a very limited range of flight, the virus is mainly spread among patches just by human mobility. In general, the spread process caused by human mobility is as follows. An individual that normally is in patch *i* (a resident) travels to patch *k*, where they becomes infected by a mosquito. Then they travels again to an other patch *j*, where a mosquito there is infected by them. The result is that the virus was spread from patch *k* to patch *j* by a resident from patch *i*. We can illustrate the generality of this with two particular cases. When *i* = *j*, it corresponds to an individual introducing the disease in their own patch because they got infected in a different patch. On the other hand, *i* = *k* represents a case when an already infected individual travel to a different patch and spread the virus in that particular patch.

The secondary cases caused by a single infected individual at a completely susceptible population, normally called the basic reproduction number *R*_0_, is one of the most important summary measures that describe the severity of an epidemic outbreak. In what follows we extend this idea to include geographical information of where the secondary cases where produced and which is the origin of the infected hosts.

First we denote $R_{kj}^{\left(v\right)}$ as the secondary human infections of residents from patch *j* produced by an infected vector in patch *k* in the disease-free state. In a similar way, we define $R_{ik}^{\left(h\right)}$ as the number of vector secondary cases in patch *k* caused by a single infected resident in patch *i*. These quantities can be hard or even impossible to be measured directly, but they may be inferred once a mathematical model for the disease is proposed.

A natural way of deriving useful risk measures from these quantities is to obtain the secondary infections $R_{i}^{\left(h\right)}$ generated by a single individual of patch *i* in the total susceptible population of the system:

$$\begin{array}{c} R_{i}^{\left(h\right)}=\sum_{j=1}^{N}\sum_{k=1}^{N}R_{ik}^{\left(h\right)}R_{kj}^{\left(v\right)}\;. \end{array}$$ (1)

Here $R_{ik}^{\left(h\right)}R_{kj}^{\left(v\right)}$ is the number of human-human secondary infections that a resident of patch *i* generated in residents of patch *j* that were produced in patch *k*. Summation in *k* is to take into account all the possible places where the contagion could take place. Summation in *j* is to account for all the secondary human infections that a single resident of *i* produces in all the system. Thus $R_{i}^{\left(h\right)}$ is a measure of the contagion capacity of residents from the patch *i*.

**Fig 1:**
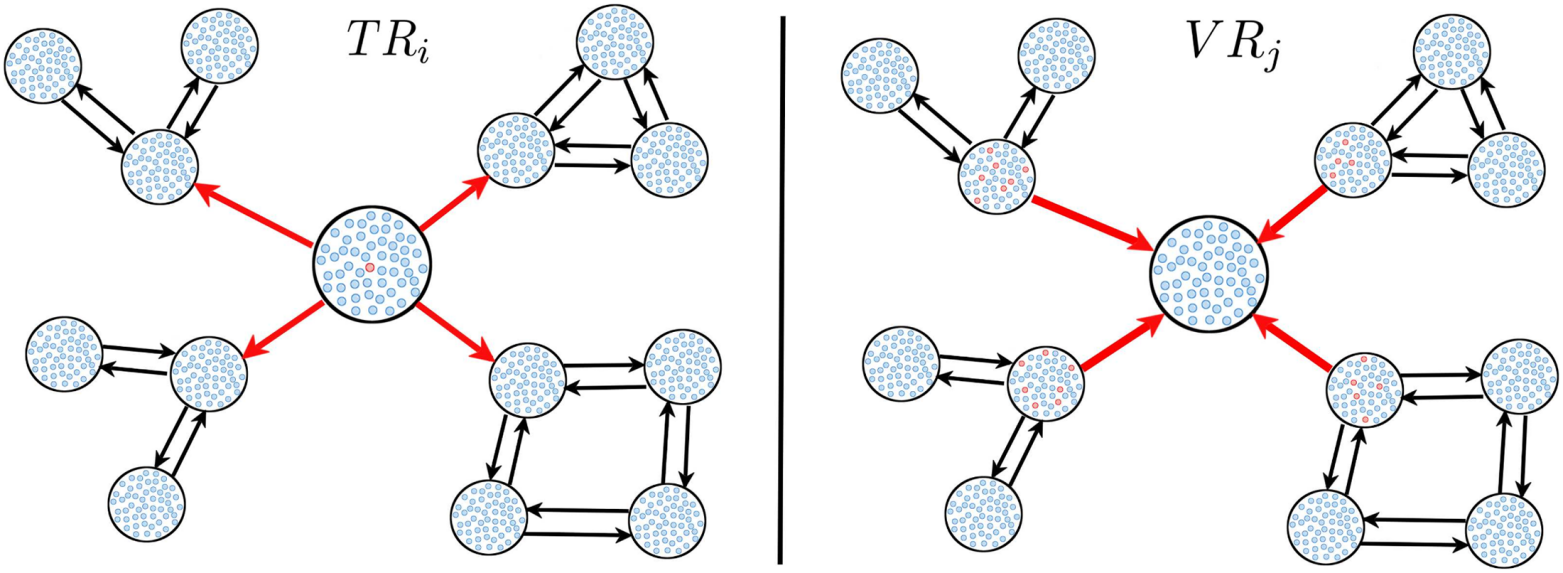
Schematic draw of a metapopulation network with human mobility. At the right we represent the measure of the Transmission Risk index (2) and, at the left, we represent the Vulnerability Risk index (4).

On the other hand, the classic definition of risk is the probability of occurrence of an unwanted event multiplied by the consequence of the event [29]. Following this definition, the transmission risk index *TR*_*i*_ is the probability *P*_*i*_ that a resident of patch *i* gets infected, multiplied by the secondary cases it generates (See Fig (1)). Thus, it is given by

$$\begin{array}{c} TR_{i}=P_{i}R_{i}^{\left(h\right)}\; \end{array}$$ (2)

and indicates the risk of the neighborhood *i* to become the main disperser of the disease at the beginning of an epidemic.

On the other hand, the number of secondary cases of patch *j* residents caused by a single infected resident of patch *i* is given by

$$\begin{array}{c} \sum_{k=1}^{N}R_{ik}^{\left(h\right)}\;R_{kj}^{\left(v\right)}\;. \end{array}$$ (3)

The summation in the previous expression accounts for the vector contagions in all patches. We can now define a Vulnerabiliy Risk index *VR*_*j*_ for patch *j* as (See Fig (1))

$$\begin{array}{c} VR_{j}=\sum_{i=1}^{N}\sum_{k=1}^{N}P_{i}\;R_{ik}^{\left(h\right)}\;R_{kj}^{\left(v\right)}\;. \end{array}$$ (4)

Finally, we define the Vector Transmission Risk index *VTR*_*i*_ as the secondary human infections $R_{i}^{\left(v\right)}$ caused by an infected vector in patch *i* multiplied by the probability of a vector of patch *i* becomes infected at the beginning of an epidemic. Then the *VTR*_*i*_ is expressed as

$$\begin{array}{c} VTR_{i}={\tilde{P}}_{i}\left(1-{\left(1-\frac{1}{w_{i}}\right)}^{N_{vi}}\right)R_{i}^{\left(v\right)}, \end{array}$$ (5)

where ${\tilde{P}}_{i}$ is the probability of the original infected host is in patch *i*, irrespective of where they came from. Also, $\left(1-{\left(1-\frac{1}{w_{i}}\right)}^{N_{vi}}\right)$ is the probability of it gets bitten by a mosquito in patch *i*, and $R_{i}^{\left(v\right)}$ is calculated as

$$\begin{array}{c} R_{i}^{\left(v\right)}=\sum_{j=1}^{N}R_{ij}^{\left(v\right)}\;. \end{array}$$ (6)

In this case $R_{i}^{\left(v\right)}$ represents the system-wide secondary human infections caused by a single infected vector in patch *i*. This index will be important just in the cases when the number of mosquitoes is large, so we can approximate it by

$$\begin{array}{c} VTR_{i}≃{\tilde{P}}_{i}\;(1-e^{-N_{vi}/w_{i}})\;R_{i}^{\left(v\right)}\;\text{for}\;N_{vi}\gg 1\;. \end{array}$$ (7)

### Model description

Our model aims to capture the dynamics of a virus transmitted by vectors (like the *Aedes aegypty* mosquito). Important viruses transmitted by this species are Dengue, Zika and Chikungunya. In order to take into account human mobility and density population within the city, we divided it into different areas or neighborhoods that we will call patches. In each patch hosts and vectors coexist, and every one has its particular social and ecological features, *i.e.* human population, transportation and mobility habits of the inhabitants, and mosquitoes density can differ from one neighborhood to other. In addition, hosts travel among patches. In this sense, patches are said to be connected.

The model is thus built in two steps. First we build a general model for a single patch, that describes the dynamics of a vector-borne disease in a single patch. After that, we extend the local model for multiple patches, representing the whole city and mobility effects.

#### Single patch model

We consider that for a given subregion Ω_*i*_, with *i* ∈ {1, 2, …, *N*}, hosts and vectors are homogeneously mixed. Furthermore, we assume host population can be classified in susceptible, infectious and recovered. On the other hand, vector population is classified in susceptible and infectious. Let *N*_*hi*_, *S*_*hi*_, *I*_*hi*_ and *R*_*hi*_ denote the number of total, susceptible, infectious and recovered host individuals, respectively, and analogously let *N*_*vi*_, *S*_*vi*_ and *I*_*vi*_ denote number of total, susceptible and infectious vector individuals respectively of patch Ω_*i*_ at a certain time. Thus, we have *N*_*hi*_ = *S*_*hi*_ + *I*_*hi*_ + *R*_*hi*_ and *N*_*vi*_ = *S*_*vi*_ + *I*_*vi*_ at any time.

In Table 1 we describe each parameter interpretation and its reference value.

**Table 1:** Description and estimated value of the model parameters ([30, 31]).

Parameter	Description	Value
*β*	Effective infectious vector biting rate (global successfully number of bites, per day, per mosquito)	[0.2, 0.67]
*β*′	Effective susceptible vector biting rate (global successfully number of bites, per day, per mosquito)	[0.2, 0.67]
*γ*	Recovery rate of hosts (per day, per capita)	1/7
*α*_*i*_	Number of eggs laid per day for every female mosquito in the *i*-th patch	5
*C*_*i*_	Carrying capacity of hatcheries for adult female mosquitoes in the *i*-th patch	[100, 10000]
*μ*_*i*_	Per-capita mortality rate of adult female mosquitoes in the *i*-th patch	1/8

For the sake of simplicity, in this subsection we will omit the patch index *i*. We consider no total hosts population change ($\dot{N_{h}}=0$), which is reasonable for a short period of time in which an outbreak occurs. On the other hand, for vectors population we use a logistic population model. The carrying capacity parameter *C* of vector population plays a major role, since it is related with the socio-environment features of the subregion that bounds vector population growth. In specific, carrying capacity is the maximal load of mosquitoes (or vectors) that environment can support in a given patch. If vital resources for the mosquitoes were unlimited, the susceptible mosquitoes *S*_*v*_ would grow at a rate *α* (ignoring mortality); however, the factor 1 − *N*_*v*_/*C* takes into account the decrease of the per-capita reproductive rate of mosquitoes due to the saturation of hatcheries.

On the other hand, the hosts population dynamics is described by a SIR type model. The coupling of both models give rise to the following system of differential equations:

$$\begin{array}{ccc} {\dot{S}}_{h} & = & -\beta I_{v}\;\frac{S_{h}}{N_{h}}\;, \end{array}$$ (8)

$$\begin{array}{ccc} {\dot{I}}_{h} & = & \beta I_{v}\;\frac{S_{h}}{N_{h}}-\gamma \;I_{h}\;, \end{array}$$ (9)

$$\begin{array}{ccc} {\dot{R}}_{h} & = & \gamma \;I_{h}\;, \end{array}$$ (10)

$$\begin{array}{ccc} {\dot{S}}_{v} & = & \alpha \;N_{v}\;(1-\frac{N_{v}}{C})-\beta^{\prime }\;S_{v}\;\frac{I_{h}}{N_{h}}-\mu \;S_{v}\;, \end{array}$$ (11)

$$\begin{array}{ccc} {\dot{I}}_{v} & = & \beta^{\prime }\;S_{v}\;\frac{I_{h}}{N_{h}}-\mu \;I_{v}\;. \end{array}$$ (12)

As usual, a susceptible vector becomes infectious when it bites an infectious host. Then, in eqs (8) and (9), *β I*_*v*_ is the total number of effective number of bites per unit of time. This bites are the ones that come from infected mosquitoes. From the total number of bites, only a portion *S*_*h*_/*N*_*h*_ affects susceptible hosts. In eqs (11) and (12), *β*′ *S*_*v*_ is the effective number of bites performed by susceptible vectors, from which only a fraction *I*_*h*_/*N*_*h*_ involve infectious hosts.

Note that in the stationary points

$$\begin{array}{c} I_{h}^{*}=I_{v}^{*}=0\;,\;N_{h}^{*}=N_{h}=S_{h}^{*}+R_{h}^{*}\;\text{and}\;N_{v}^{*}=S_{v}^{*}=\tilde{C}\;, \end{array}$$

where $\tilde{C}=\left(1-\left(\mu /\alpha \right)\right)\;C$ is the effective carrying capacity.

**Fig 2:**
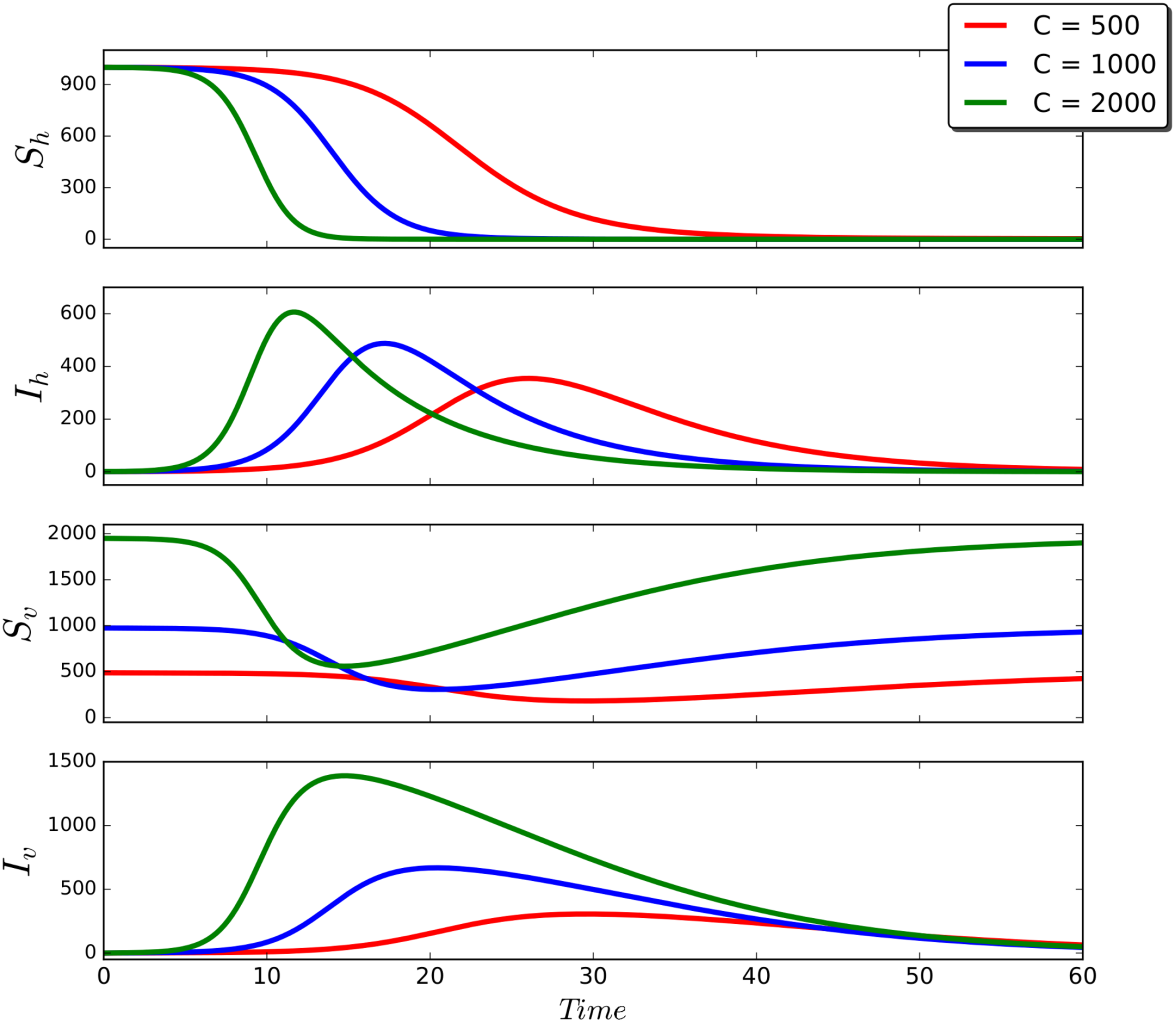
Time series of the model (8)–(12) for different values of the carrying capacity *C*, and the parameters values given in Table 1 with *β* = 0.67.

In order to illustrate the vector-borne disease dynamics for a single patch, we show in Fig 2 the time series of each one of the variables of the system (8)–(12) by selecting the parameter values given in Table 1. In regards of the carrying capacity parameter *C*, we select the following values: 500, 1000 and 2000. For each of these ones, we select as initial condition of the system the values $\left[S_{ho},I_{ho},R_{ho},S_{vo},I_{vo}\right]=\left[1000,1,0,\tilde{C},0\right]$.

#### Multiple connected patches model with hosts dwell time

In this section we extend the local model described by eqs (8)–(12) to the setting of multiple connected patches. Human mobility among distinct patches of Ω play an important role in the propagation of infectious diseases, and specifically of vector-borne ones. Consider an scenario where resident hosts of the *i*-th patch spend a period of their day (relevant with respect to vector activity) over the *j*-th patch. If there are infectious vectors in patch *j*, and some of them bite someone of these susceptible visitants, then these human hosts will bring the disease to patch *i*. In this way, some vectors in patch *i* may also get infected, which at the same time will potentially infect, in subsequent days, both resident hosts in the *i*-th patch and visitors coming from other patches. On the other hand, infected visitants from patch *i* could infect susceptible vectors in patch *j* in case they were bitten, producing same result in visited patch *j*.

Consequently, realistic models of vector-borne diseases spread demand a deep enough comprehension of this phenomena. In “small” regions, as cities are, human mobility among neighborhoods is usually repetitive and frequent. For instance, people go to work and return home almost every day, or go to the cinema or visit a friend, and then come back home in the same day. Furthermore, most of urban mobility within a city is by far caused by day-trippers. It is also very important (as far as applications are concerned) that this kind of motion follows patterns that can be measured, predicted or estimated.

There have been two distinct approaches in the literature in order to model the effects of mobility in epidemiology; the Lagrangian and the Eulerian approach. The Lagrangian approach tracks the visits effect without explicitly modeling the flux of people. This approach is more natural applied when modeling short term repetitive mobility patterns like the ones previously mentioned for a city. The Eulerian approach describes the actual flux of people. This approach is useful in modeling any kind of migration.

We are interested in repetitive day-trippers mobility patterns, thus it is convenient to follow a Lagrangian approach. We introduce a set of parameters ${\left\{p_{ij}\right\}}_{i,j=1}^{N}$ with the following meaning: for each pair *i* and *j* with 1 ≤ *i* ≤ *N* and 1 ≤ *j* ≤ *N*, *p*_*ij*_ is simultaneously (see the S1 Appendix):

- The average fraction of people from patch *i* that is in patch *j* at any time. That means that the number of individuals from patch *i* who are in patch *j* is *p*_*ij*_ *N*_*hi*_, at any time.
- The probability, at any time, to find a host from patch *i* in patch *j*.
- The average fraction of time (a day or the corresponding time unit measure) that people from patch *i* spend in patch *j*. That is why it is also called host dwell time [21].

Furthermore, as *p*_*ij*_ represent fractions we have the following conditions on mobility parameters:

$$\begin{array}{c} 0\leq p_{ij}\leq 1\;\text{and}\;\sum_{j=1}^{N}p_{ij}=1\;. \end{array}$$

In terms of this quantities, the number of individuals who are in patch *j* is given by

$$\begin{array}{c} w_{j}=\sum_{i=1}^{N}p_{ij}\;N_{hi} \end{array}$$ (13)

at any time *t*, regardless the patch where they are coming from (that is, including both own residents and day-trippers).

Now we are ready to write the global model of multiple connected patches. Starting from the local model, namely (8)–(12), we couple each local dynamic taking into account human mobility in the terms discussed before, obtaining the following system of 5*N* differential equations: for each *i* = 1, …, *N*

$$\begin{array}{ccc} {\dot{S}}_{hi} & = & -\sum_{j=1}^{N}\beta \;I_{vj}\;\frac{p_{ij}\;S_{hi}}{w_{j}}\;, \end{array}$$ (14)

$$\begin{array}{ccc} {\dot{I}}_{hi} & = & \sum_{j=1}^{N}\beta \;I_{vj}\;\frac{p_{ij}\;S_{hi}}{w_{j}}-\gamma \;I_{hi}\;, \end{array}$$ (15)

$$\begin{array}{ccc} {\dot{R}}_{hi} & = & \gamma \;I_{hi}\;, \end{array}$$ (16)

$$\begin{array}{ccc} {\dot{S}}_{vi} & = & \alpha_{i}\;N_{vi}\;(1-\frac{N_{vi}}{C_{i}})-\sum_{j=1}^{N}\beta^{\prime }\;S_{vi}\;\frac{p_{ji}\;I_{hj}}{w_{i}}-\mu_{i}\;S_{vi}\;, \end{array}$$ (17)

$$\begin{array}{ccc} {\dot{I}}_{vi} & = & \sum_{j=1}^{N}\beta^{\prime }\;S_{vi}\;\frac{p_{ji}I_{hj}}{w_{i}}-\mu_{i}\;I_{vi}\;. \end{array}$$ (18)

We proceed to explain the above equations. With respect of eqs (14) and (15), for a fixed *i*, for each *j* with 1 ≤ *j* ≤ *N*, *β I*_*vj*_ is the total number of bites of infectious vectors to hosts per day in patch *j*. From which, only a portion are on susceptible ones to become sick in the *i*-th patch. Since *p*_*ij*_ is the average fraction of people from patch *i* in patch *j*, and assuming this average is independent of the susceptible or infectious populations, then *p*_*ij*_ *S*_*hi*_ is the number of susceptible hosts from patch *i* who are in patch *j*. Thus, if *w*_*j*_ is the number of people in patch *j*, the portion of susceptible hosts from patch *i* among people in patch *j* is *p*_*ij*_ *S*_*hi*_/*w*_*j*_. According to these interpretations, *β* *I*_*vj*_ *p*_*ij*_ *S*_*hi*_/*w*_*j*_ is the rate of new infected hosts from patch *i* being infected in patch *j*. Finally, we add over all patches *j* to get the total number of infected hosts from patch *i*.

Analogously, in eqs (17) and (18), *β*′ *S*_*vi*_ is the number of bites performed by susceptible vectors in patch *i*, *p*_*ji*_ *I*_*hj*_ is the number of infective human visitors of patch *i* coming from patch *j*, and *p*_*ji*_ *I*_*hj*_/*w*_*i*_ is the fraction of infectious hosts from patch *j* in patch *i*. Consequently, *β*′ *S*_*vi*_ *p*_*ji*_ *I*_*hj*_/*w*_*i*_ is the number of infected vectors per unit time in patch *i* caused by human hosts from patch *j*. Finally, we add over all patches *j* to get the total number of infected vectors in patch *i*.

The entire mobility configuration is determined by the *N* × *N* coupling matrix $P={\left(p_{ij}\right)}_{i,j=1}^{N}$, whose entry corresponding to the row *i* and column *j* is *p*_*ij*_. *P* is a right stochastic matrix (its entries are probabilities and its rows sum one) non necessarily symmetric (*i.e.* in general *p*_*ij*_ ≠ *p*_*ji*_).

**Fig 3:**
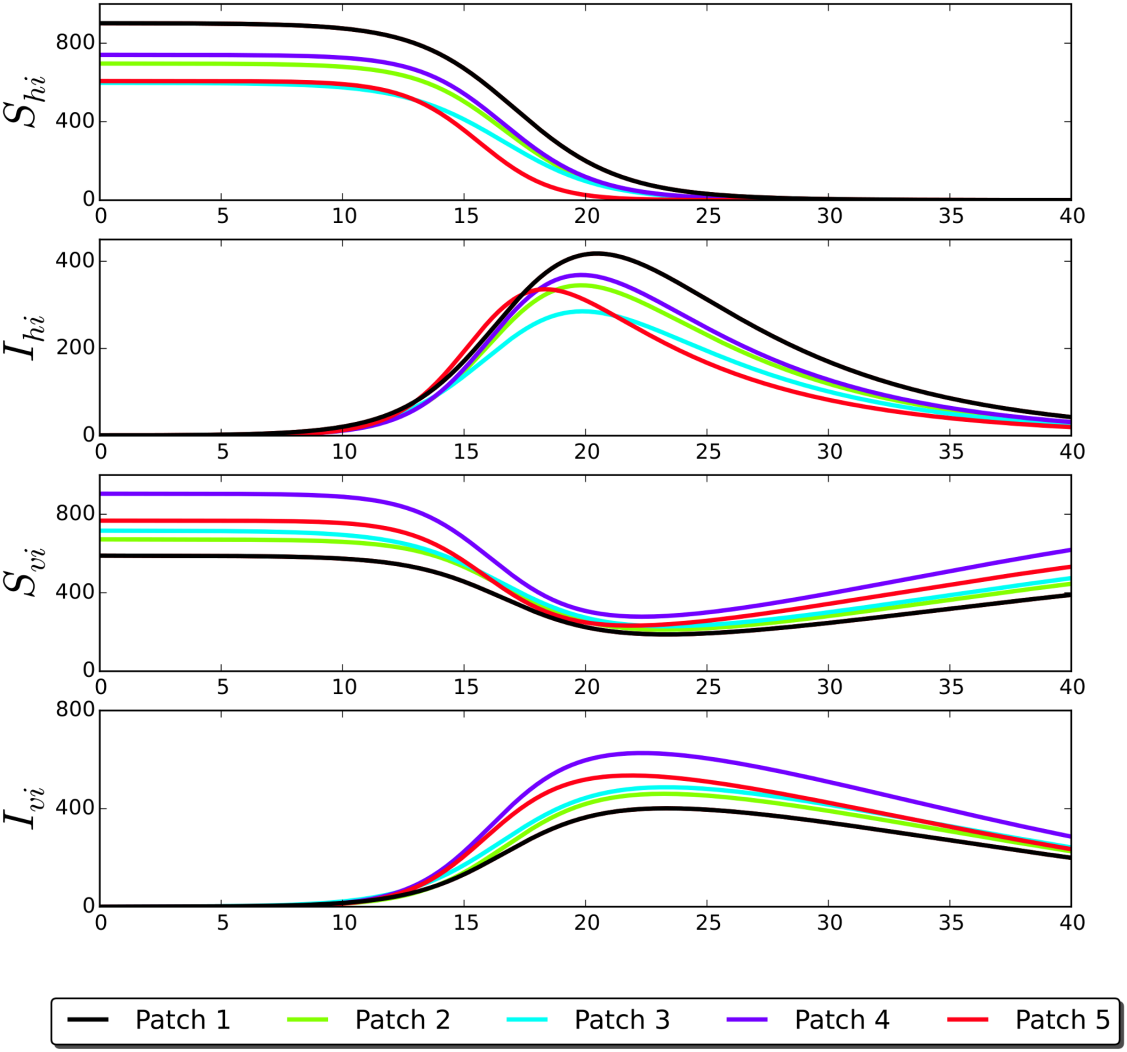
Time series of the multiple patch connected according to the dwell-time matrix *P*_*e*.*g*.1_ given in (19).

By way of example, we perform a numerical simulation (See Fig (3)) for a network of *N* = 5 patches coupled in the following configuration:

$$\begin{array}{c} P_{e.g.1}=(\begin{array}{ccccc} 0.4143 & 0.2922 & 0.2934 & 0 & 0\\ 0.3340 & 0.3597 & 0 & 0.3061 & 0\\ 0.3773 & 0 & 0.6226 & 0 & 0\\ 0 & 0.6658 & 0 & 0.1307 & 0.2034\\ 0 & 0 & 0 & 0.3954 & 0.6045 \end{array})\;. \end{array}$$ (19)

For this example we chose, uniform randomly, the dwell-time parameters *p*_*ij*_ (for *i*, *j* = 1, …, 5), the same parameters values given in Table 1 for all the patches but we vary the carrying capacity taken from a power law probability distribution in the range *C*_*i*_ ∈ [100, 1000]. It is also worth to mention that we select the connection pattern configuration described by the matrix (19) in order to emulate, as close as possible, a scenario where some patches are most visited than another ones. Based on the empirical Taylor’s power law [32] from ecology, that relates the variance of the number of individuals per unit area to the corresponding mean by a power law relationship. We select the carrying capacity from a power law distribution, as this kind of population clustering has been observed in populations of *Aedes aegypti* [33]. In regards of the initial conditions, we assume that at the initial time there are not infectious host and vectors; *i.e.* *I*_*hi*_(*t* = 0) = *I*_*vi*_(*t* = 0) = 0 for almost all values of the index *i*. In this example we select randomly a single patch and we introduce an infected host, that is *I*_*hj*_(*t* = 0) = 1 for a given randomly selected patch *j*. The initial number of susceptible hosts *S*_*hi*_(*t* = 0) ∈ [500, 1000] were taken from a power law distribution in order to imitate a metapopulation network with some patches that condense a great number of residents like habitational neighborhoods compared to the other patches representing commercial or low density neighborhoods. *S*_*vi*_(*t* = 0) = *C*_*i*_(1 − *μ*_*i*_/*α*_*i*_) for all *i* and the rest of the variables start at zero.

## Results

### Expressions for the indexes

In order to find an explicit expression for the secondary human cases $R_{kj}^{\left(v\right)}$ caused by a single infected mosquito in patch *k*, we have to multiply the rate of infections generated from mosquitoes of patch *k* to visitors from patch *j*, i.e. $\frac{\beta \;S_{hj}\;p_{jk}\;I_{vk}}{w_{k}}$ times the characteristic duration time that a mosquito remains infected, 1/*μ*_*k*_. Then we evaluate this quantity with a single infected vector in the disease-free equilibrium. That is

$$R_{kj}^{\left(v\right)}={\frac{\beta \;S_{hj}\;p_{jk}\;I_{vk}}{\mu_{k}\;w_{k}}|}_{S_{hj}=N_{hj},I_{vk}=1}=\frac{\beta N_{hj}p_{jk}}{\mu_{k}w_{k}}.$$ (20)

In a similar way, from the proposed model an explicit expression for secondary vector infections in patch *k* that were caused by travelers from patch *i* at the beginning of an epidemic is

$$\begin{array}{c} R_{ik}^{\left(h\right)}=\frac{\beta^{\prime }\;p_{ik}\;N_{vk}}{\gamma w_{k}}\;. \end{array}$$ (21)

Thus, $R_{i}^{\left(h\right)}$ is given by

$$\begin{array}{c} R_{i}^{\left(h\right)}=\sum_{j=1}^{N}\sum_{k=1}^{N}R_{ik}^{\left(h\right)}\;R_{kj}^{\left(v\right)}=\frac{\beta \;\beta^{\prime }}{\gamma }\;\sum_{k=1}^{N}\frac{p_{ik}\;N_{vk}}{\mu_{k}\;w_{k}}\;. \end{array}$$ (22)

If we assume that each person in the system has equal chance of starting the outbreak, then we have a particular case where *P*_*i*_ = *N*_*i*_/*N*_*h*_, and the transmission risk index becomes

$$\begin{array}{c} TR_{i}=\frac{N_{hi}}{N_{h}}\;R_{i}^{\left(h\right)}=\frac{\beta \;\beta^{\prime }\;N_{hi}}{\gamma N_{h}}\;\sum_{k=1}^{N}\frac{p_{ik}\;N_{vk}}{\mu_{k}w_{k}}\;. \end{array}$$ (23)

In a similar way, the vulnerability index takes the form

$$\begin{array}{c} VR_{j}=\sum_{i=1}^{N}\sum_{k=1}^{N}\frac{N_{hi}}{N_{h}}\;R_{ik}^{\left(h\right)}\;R_{kj}^{\left(v\right)}=\frac{\beta \;\beta^{\prime }\;N_{hj}}{\gamma N_{h}}\;\sum_{k=1}^{N}\frac{p_{jk}N_{vk}}{\mu_{k}w_{k}}\;, \end{array}$$ (24)

and finally the vector transmission risk is

$$\begin{array}{c} VTR_{i}≃\frac{w_{i}}{N_{h}}\;(1-e^{-N_{vi}/w_{i}})\;R_{i}^{\left(v\right)}\;, \end{array}$$ (25)

where

$$\begin{array}{c} R_{i}^{\left(v\right)}=\sum_{j=1}^{N}R_{ij}^{\left(v\right)}=\frac{\beta }{\mu_{i}}\;, \end{array}$$ (26)

and we have taken the particular case where ${\tilde{P}}_{i}=\frac{w_{i}}{N_{h}}$. For the sake of simplicity, we consider this simple case. However, other probability distributions which take into account well known factors of introduction of vector-borne diseases into a population, as importation by travellers or some trading businesses, could be considered at this point.

### Numerical simulations

In order to show how the risk indexes can be used to guide control strategies we simulate an ensemble of Dengue outbreaks using the model (14)–(18). First we perform an ensemble of 200 simulations with parameter values shown in Table 1. For each simulation, we choose randomly the initial infected host individual. We used 5 patches and the number of humans and carrying capacity in each patch was taken randomly in the range 4000-10000 and 1000-2800 respectively. See Table 2 for the particular values. We used two types of mobility matrix: the first is an unrestricted mobility, where any average fraction of people from patch *i* can go to any other patch *j*; the second type of tested mobility matrix resembles a network constructed with the Barabasi-Albert algorithm [34], in which there exist patches that are more visited than others. The particular mobility matrices used in the first and second type of mobility are labeled with *P*_1_ and *P*_2_, respectively, and its corresponding values are showed in the S1 Appendix.

**Table 2:** Carrying capacity *C*_*i*_ and human population *N*_*hi*_ used in the simulations within each patch. *R*_0_ if patches where isolated and risk index values for two types of networks.

Patch	*C*_*i*_	*N*_*hi*_	*R*_0_	Unrestricted mobility			Barabasi-Albert mobility		
				*TR*_*i*_	*VR*_*i*_	*VTR*_*i*_	*TR*_*i*_	*VR*_*i*_	*VTR*_*i*_
1	1435.90	6587	2.31	0.92	0.22	0.19	**1.70**	0.22	0.19
2	2564.10	7292	2.93	1.30	**0.42**	**0.52**	1.13	0.39	0.39
3	1025.64	9791	1.60	**1.68**	0.16	0.22	1.18	0.11	0.12
4	2051.28	5875	2.92	1.07	0.33	0.26	1.01	**0.43**	**0.46**
5	1025.64	4354	2.40	0.86	0.15	0.08	0.81	0.14	0.12

**Fig 4:**
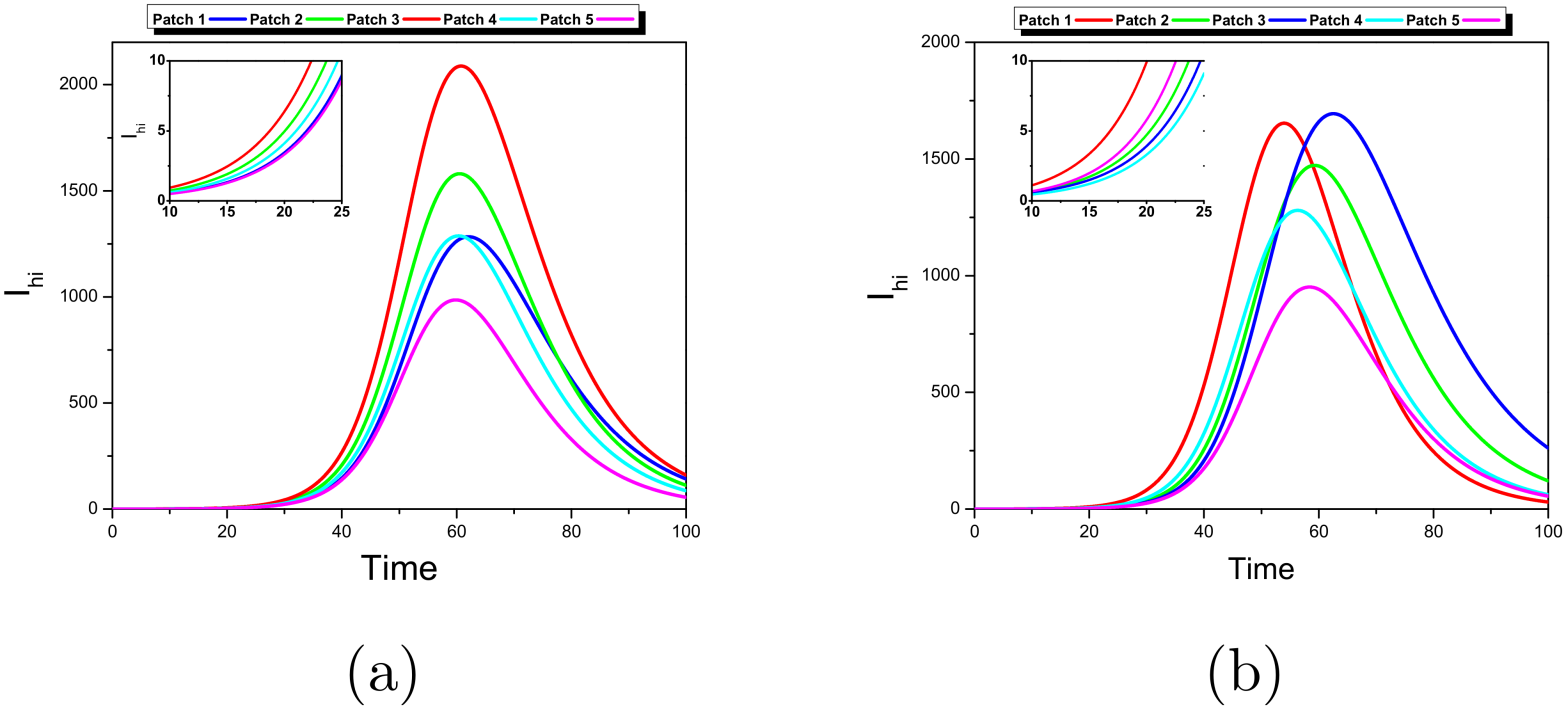
Dynamics of the number of infected humans per patch in a network of 5 nodes for a fully connected network (a) and a Barabasi-Albert network (b). In (a) the vulnerability index *VR*_*i*_ indicates patch 3 (red line) as the most vulnerable and in (b) the most vulnerable as indicated by *VR*_*i*_ is patch 1 (red line). The most vulnerable patch corresponds to the one with more cases at the beginning of the epidemic (let say *t* ≤ 50).

According to the definition, the vulnerability index *VR*_*i*_ stands for the expected number of secondary human cases in patch *i* produced in a totally susceptible population by an initial sick host, assuming we do not know where they comes from. Consequently, this index gives an idea of the severity of the epidemic in the corresponding patch at the very beginning of it: the more large this index is, the more great the number of infected hosts will be in this patch. Concretely, the patch with greatest vulnerability index *VR*_*i*_ corresponds to the patch with more infections at the first stages of the outbreak, as can be seen in Fig 4 for the above types of mobility. Note that this picture could change as long as the epidemic evolves to a different state from the initial totally susceptible one.

We then compare the effect of applying an specific control measure in any other patch (randomly selected, for instance) with the effect of applying the same control measure but in the patch indicated by an adequate index (in relation to the control measure).

**Fig 5:**
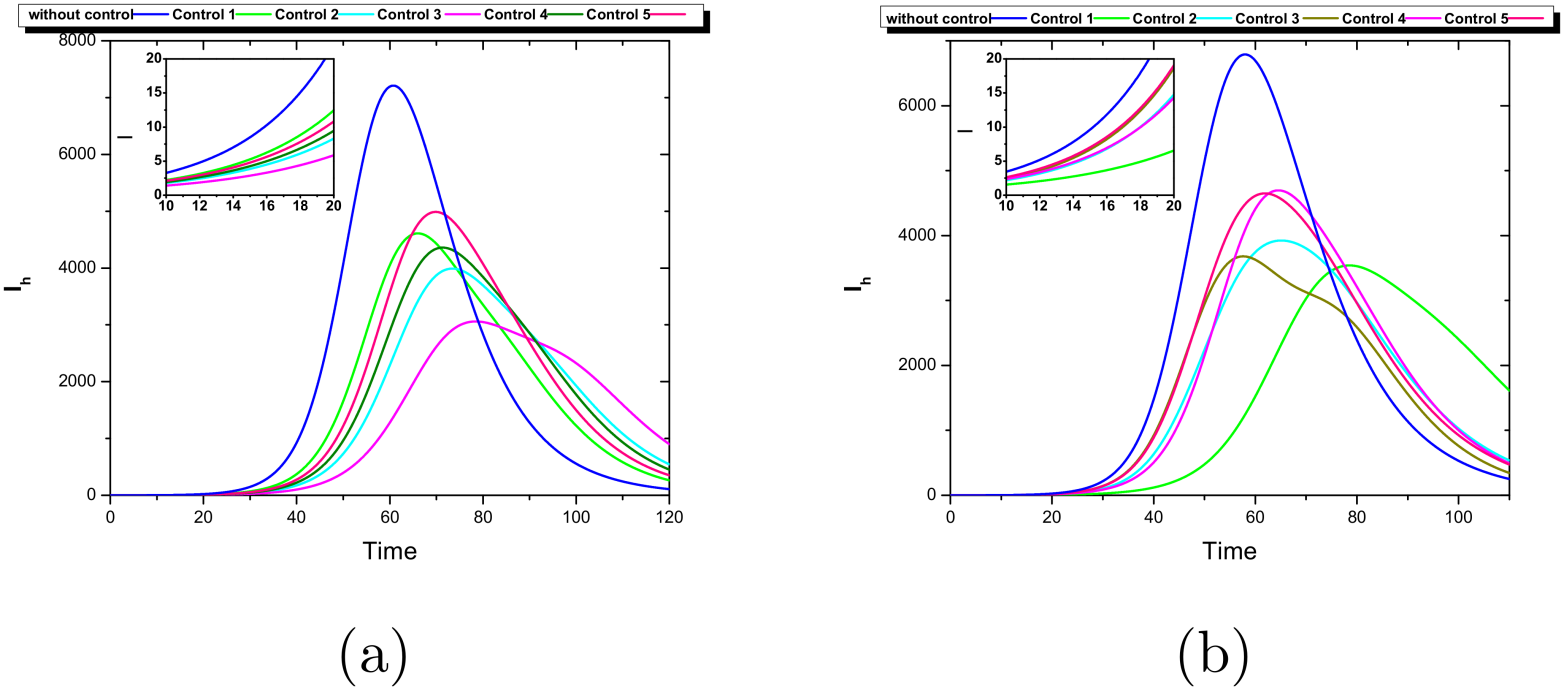
Dynamics of the infected population system-wide with control applied in different patches. (a) In a fully connected network the transmission index *TR*_*i*_ is greater in patch 3. (b) In a Barabasi-Albert network the patch with more transmissibility is number 1 as indicated by its *TR*_*i*_. The most effective strategy is to reduce the infected hosts (by treatment and hospitalization) in the patch with greatest *TR*_*i*_.

Following the risk indexes definition, the transmission risk index *TR*_*i*_ measures the transmission power of a given patch; that is, the secondary cases an initial sick host in patch *i* produces in a complete totally susceptible population, weighted by the probability of patch *i* being the initial focus of the epidemics. Then, the patch with highest transmission risk index *TR*_*i*_ is the candidate to become the main disperser of the disease at the fist stage of the epidemics. This index can be linked with control measures on human populations. Therefore, the first tested control measure was the fast isolation of infected people by hospitalizing them as soon as an infection is detected. We simulate this by increasing the recovery rate *γ* of a single patch. Fig 5 shows, as expected, that this measure is more effective when applied in the patch with greatest transmission index *TR*_*i*_ than in any other patch, on average. One can see in Fig 5 that the amount of total infected hosts *I*_*h*_ is the lowest one for *t* ≤ 50 when the disease control measure is applied to the patch with largest transmission index *TR*_*i*_.

**Fig 6:**
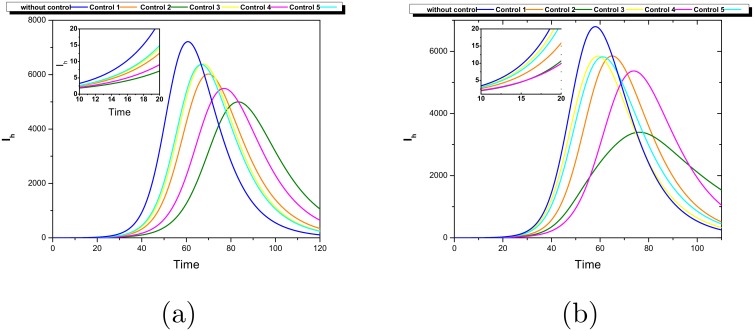
Dynamics of the infected population system-wide with control applied in different patches. (a) In a fully connected network the vector transmission index *VTR*_*i*_ is greater in patch 2. (b) In a Barabasi-Albert network the patch with largest vector transmissibility is number 4 as indicated by its *VTR*_*i*_. The most effective strategy at the beginning of the outbreak is to reduce the carrying capacity in the patch with greatest *VTR*_*i*_.

Finally, analogously to the previous one, the vector transmission risk index *VTR*_*i*_ measures the transmission power of a given patch taken into account its vector population; that is, the secondary human cases an initial infectious vector in patch *i* produces in a complete totally susceptible population, weighted by the probability of patch *i* being the initial focus of the epidemics, wherever the initial carrier host come from. Hence, the patch with largest vector transmission risk index *VTR*_*i*_ is the candidate to become initially the main disperser of the disease at the beginning of the epidemics, regarding to the vector dynamics. This index can be linked with control measures on vector populations. Accordingly, we consider here the reduction of the local vector population as the control measure (resulting from abatization, fumigation, hatchery elimination…). We simulate this by reducying the carrying capacity *C*_*i*_ (or directly by removing the local vector population) of a single patch. As expected, the vector transmission risk index *VTR*_*i*_ was able to indicate the patch where this strategy proved to be more effective to reduce the initial growth of the epidemic, on average (see Fig 6). That is, the amount of total infected hosts *I*_*h*_ is the lowest one for *t* ≤ 50 when the control is applied to the patch with highest vector transmission risk index *VTR*_*i*_, compared to the other patches located control strategy, and to the unchecked situation.

## Discussion

We propose three risk indexes for the transmission of vector-borne diseases. Two of them are related with the transmission risk of patches and the other one gives a measure of the vulnerability of each patch. It is worth to notice their ability to localize epidemiological risk at different nodes of a net.

The transmission indexes of a patch quantify the potential secondary cases that a first infected individual or vector in this patch may generate system-wide.

On the other hand, the vulnerability index of a patch quantify the potential secondary cases that a first infected individual in the system cause in the relevant patch.

Specifically, the transmission risk index *TR*_*i*_ is the probability of a host in patch *i* became the first infected host, times secondary human infections they would potentially cause. The second index *VTR*_*i*_, called vector transmission risk index, is defined as the probability of a vector gets first infected in patch *i*, times secondary human infections it would potentially cause. The vulnerability index *VR*_*i*_ is secondary host infections in patch *i* averaged over a randomly distributed initial infected human in the system.

These risk indexes can be used in different ways. First, they indicate optimal places to apply prevention measures. They are also useful to guide the monitoring campaigns for an early detection of epidemics. And finally, they can guide control and mitigating strategies at the beginning of an outbreak.

For example, the transmission risk index *TR*_*i*_ indicate that the patch with the greatest value of it, is the best place to distribute repellents as a prevention strategy. Another control strategy is to isolate by hospitalization infected individuals of such a neighborhood.

The vector transmission risk *VTR*_*i*_ indicates the most suitable places where abatization and fumigation campaigns will be more effective. Thus, when there are limited resources, the best strategy is to give priority to the patches with higher values of the index. On the other hand, the vulnerability risk index *VR*_*i*_ can guide investments in health clinics. This would increase the public health capacity in particular areas where many cases will likely arise. In addition to this, as it is expected to find more cases in the neighborhood with highest *VR*_*i*_, this is a good place for incidence monitoring. That will help in detecting increased anomalous endemic states or an outbreak.

In order to illustrate these control measures, we compared a set of simulations in the frame of a proposed particular model for vector-borne diseases spread in a multi-patch system for a Dengue epidemics. In addition to vector and host densities, this model takes into account human mobility. We have observed that an immediate medical attention gives benefits to the whole population if the neighborhood with highest transmission risk *TR*_*i*_ is prioritized. In contrast, reducing the hatcheries for example by abatization brings more benefits if the neighborhood with highest vector transmission risk *VTR*_*i*_ is prioritized. We observed in the simulations that the risk indexes can also be used to design control strategies but just at the beginning of the epidemic. The time these indexes give the best strategy during an epidemic depends on the network structure. A complete study of the relationship among all possible network configurations, the corresponding risk indexes values, and the most efficient strategies is out of the scope of the current work. In order to apply these indexes in practical situations, it is necessary to have estimations of the model parameters. With respect to this, entomological parameters can be estimated using reported values in the literature at different temperatures [35, 36]. On the other hand, the now ubiquitous tracking technologies based on mobile devices such as google maps, twitter of even traffic cams, have opened the possibility to estimate with high resolution human mobility patterns. Finally, even if the carrying capacity of each patch is difficult to measure, to stratify a region it is not necessarily to know its absolute value. It is just enough to know the relative importance of it among the patches. This can be obtained by the usual field work carried by the entomologist.

## Supporting information

S1 Appendix Concerning host dwell times. (PDF)
