# Supplementary material for: Vector-borne disease risk indexes in spatially structured populations
Source: PLoS Negl Trop Dis. 2018 Feb 12;12(2):e0006234. doi: 10.1371/journal.pntd.0006234 (PMC5825167; doi:10.1371/journal.pntd.0006234)
Supplement: S1 Appendix — (PDF) [file pntd.0006234.s001.pdf]

S1 Appendix - Concerning host dwell times

In this paper we use two types of dwell-time matrices in order to show how the risk indexes can be used in a metapopulation network. In the first type, we assume that the network topology correspond to a fully connected graph where the residents of a given patch can travel to any other. In specific, we use the following dwell-time matrix:

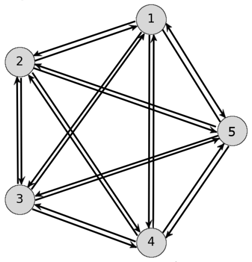

$$P_1 = \begin{pmatrix} 0.2967 & 0.0765 & 0.1650 & 0.0642 & 0.3973 \\ 0.1840 & 0.1304 & 0.2441 & 0.2919 & 0.1495 \\ 0.2283 & 0.1299 & 0.1900 & 0.2244 & 0.2271 \\ 0.0843 & 0.1977 & 0.2613 & 0.2146 & 0.2418 \\ 0.2609 & 0.2294 & 0.1724 & 0.2063 & 0.1307 \end{pmatrix}$$

where the entries p_{ij} (for $i, j = 1, \dots, N$) of the matrix P_1 are selected randomly with uniform probability. On the other hand, the second type of dwell-time matrix P_2 is constructed by using the Barabasi-Albert algorithm in order to guarantee the existence of patches that are more visited than others. The values of the entries of P_2 are selected randomly with uniform probability; its specific values are:

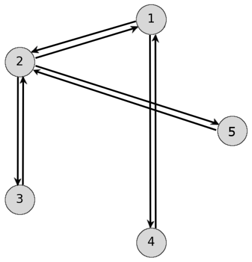

$$P_2 = \begin{pmatrix} 0.2127 & 0.2633 & 0 & 0.5238 & 0 \\ 0.2675 & 0.2175 & 0.1698 & 0 & 0.3450 \\ 0 & 0.2074 & 0.7925 & 0 & 0 \\ 0.7656 & 0 & 0 & 0.2343 & 0 \\ 0 & 0.3662 & 0 & 0 & 0.6337 \end{pmatrix}$$

Let us clarify the meaning of parameters $\{p_{ij}\}_{i,j=1}^{N}$. Fix an index i with $1 \leq i \leq N$, and consider a typical human individual of patch i. Assign them provisionally a second index k, with $1 \leq k \leq N_{hi}$. Their motion can be tracked down with a function $\phi_{i,k} : [0, \infty) \longrightarrow \{1, \dots, N\}$ in the following way: for any $t \geq 0$ and j $(1 \leq j \leq N)$ $\phi_{i,k}(t) = j$ means that individual k from patch i is in patch j at time t. Denote $\Phi_{i,j}(t)$ the number of human individuals of patch i who are in patch j at time t (number of indexes k such that $\phi_{i,k}(t) = j$).

If the population is large enough and their mobility habits are repetitive, it makes sense to assume that, in spite of functions $\phi_{i,k}$ depend on time, quantities $\Phi_{i,j}$ actually do not. Denote $p_{ij} = \Phi_{i,j}/N_{hi}$ for any pair i, j. Equipped with this convention, clearly p_{ij} is the average fraction of people from patch i in patch j at any time, and $\Phi_{i,j} = p_{ij} N_{hi}$ is the number of individuals from patch i who are in patch j, at any time. On the other hand, given an individual from patch i (given k with $1 \leq k \leq N_{hi}$), the probability to find this host in some particular patch j is the probability of having $\phi_{i,k}(t) = j$. Assuming this one to be independent of the particular k (population is homogeneously mixed at every single patch), this quantity is obviously number of favorable cases over

total cases, *i.e.* $\Phi_{i,j}/N_{hi} = p_{ij}$.

The third interpretation, in terms of dwell times, is a little more involved. Let's denote χ_T the indicator function of the set T (*i.e.* $\chi_T(t) = 1$ if $t \in T$ and $\chi_T(t) = 0$ elsewhere), and $|T|$ the length (or Lebesgue linear measure) of T. Suppose functions $\phi_{i,k}$ are simultaneously τ-periodic (as mobility habits are repetitive) for some $\tau > 0$ (the time unit measure). Thus $\phi_{i,k} = \sum_{j=1}^{N} j\, \chi_{T_{ijk}}$ (restricted to its period) for some sets $\{T_{ijk}\}$ in $[0, \tau]$. This is equivalent to say that $\phi_{i,k}(t) = j$ iff $t \in T_{ijk}$, so $|T_{ijk}|/\tau$ is the fraction of time that individual k from patch i spends in patch j in any period. Also, we find that

$$\Phi_{i,j}(t) = \sum_{\substack{k=1 \\ \phi_{i,k}(t)=j}}^{N_{hi}} 1 = \sum_{k=1}^{N_{hi}} \chi_{T_{ijk}}(t)\,.$$

Thus, finally, the average fraction of time that people from patch i spend in patch j is

$$\frac{1}{N_{hi}} \sum_{k=1}^{N_{hi}} \frac{|T_{ijk}|}{\tau} = \frac{1}{N_{hi}\,\tau} \int_0^{\tau} \Phi_{i,j}(t)\, dt = \frac{p_{ij}\, N_{hi}\, \tau}{N_{hi}\, \tau} = p_{ij}\,.$$

Furthermore, we have the following conditions on mobility parameters. Clearly $0 \le \Phi_{ij} \le N_{hi}$ and $\sum_{j=1}^{N} \Phi_{i,j}(t) = N_{hi}$, so we have

$$0 \le p_{ij} \le 1 \qquad \text{and} \qquad \sum_{j=1}^{N} p_{ij} = 1\,,$$

and we also find that the number of individuals who are in patch j is

$$w_j = \sum_{i=1}^{N} \Phi_{i,j} = \sum_{i=1}^{N} p_{ij}\, N_{hi}$$

at any time t, regardless the patch where they are coming from (that is, including both own residents and day-trippers).Of course, this way of reasoning is merely motivational. We admit any real value between zero and one for p_{ij}, and not only $p_{ij} = \nu/N_{hi}$, with ν, N_{hi} integers fulfilling $0 \le \nu \le N_{hi}$.
